# Phenotypic Discovery of Thiocarbohydrazone with Anticancer Properties and Catalytic Inhibition of Human DNA Topoisomerase IIα

**DOI:** 10.3390/ph16030341

**Published:** 2023-02-23

**Authors:** Ilija N. Cvijetić, Barbara Herlah, Aleksandar Marinković, Andrej Perdih, Snežana K. Bjelogrlić

**Affiliations:** 1 Faculty of Chemistry, University of Belgrade, Studentski trg 12-16, 11000 Belgrade, Serbia; 2 National Institute of Chemistry, Hajdrihova 19, SI 1000 Ljubljana, Slovenia; 3 Faculty of Pharmacy, University of Ljubljana, Aškerčeva 7, SI 1000 Ljubljana, Slovenia; 4 Faculty of Technology and Metallurgy, University of Belgrade, Karnegijeva 4, 11120 Belgrade, Serbia; 5 National Cancer Research Center, Pasterova 14, 11000 Belgrade, Serbia

**Keywords:** thiocarbohydrazones, human DNA topoisomerase IIα, catalytic inhibitors, dynophores, molecular dynamics, cancer research, phenotypic screening

## Abstract

Phenotypic screening of α-substituted thiocarbohydrazones revealed promising activity of 1,5-bis(salicylidene)thiocarbohydrazide against leukemia and breast cancer cells. Supplementary cell-based studies indicated an impairment of DNA replication via the ROS-independent pathway. The structural similarity of α-substituted thiocarbohydrazone to previously published thiosemicarbazone catalytic inhibitors targeting the ATP-binding site of human DNA topoisomerase IIα prompted us to investigate the inhibition activity on this target. Thiocarbohydrazone acted as a catalytic inhibitor and did not intercalate the DNA molecule, which validated their engagement with this cancer target. A comprehensive computational assessment of molecular recognition for a selected thiosemicarbazone and thiocarbohydrazone provided useful information for further optimization of this discovered lead compound for chemotherapeutic anticancer drug discovery.

## 1. Introduction

Cancer is the second leading cause of death worldwide, and, according to the World Health Organization (WHO), it was responsible for nearly 10 million deaths in 2020, or nearly one in six deaths [1]. It comprises a diverse group of diseases that result from abnormal cell growth and can potentially invade and metastasize to other parts of the body. One of the hallmarks of cancer is rapid, uncontrolled cell proliferation [2,3], and the inhibition of this process has been the focus of cancer research since its inception, resulting in many efficient chemotherapy regimens [4].

Rapidly dividing cancer cells require the enhanced activity of a family of DNA topoisomerases, efficient biological nanomachines that catalyze formation of either transient single-strand breaks (type I topoisomerases, topo I) or double-strand breaks (type II topoisomerases, topo II) and regulate the topological changes of the DNA molecule. An important member of this family is the human topoisomerase IIα, an ATP-dependent enzyme [5] that exists in α and β isoforms [6] and shares approximately 70% sequence similarity but is differentially regulated during cell growth. Topo IIα is elevated in proliferating cells, whereas topo IIβ is present in proliferating as well as postmitotic cells. Human topo IIα represents a main target of the type II family for cancer therapies and is targeted by many established anticancer drugs such as etoposide, doxorubicin, daunorubicin, and mitoxantrone [7,8]. These compounds are classified as topoisomerase poisons because they exert anticancer activity by stabilizing the transient covalent DNA–topo II complex, which blocks DNA replication and transcription and promotes cell apoptosis. Although they are clinically highly efficient, the use of topo II poisons is limited by rapidly developing cancer resistance and severe side effects such as the induction of secondary malignancies and cardiotoxicity [7,9,10,11]. To avoid these side effects, an emerging group of catalytic topo II inhibitors was extensively studied [12]. These compounds interfere with the catalytic cycle of topo II without inducing DNA damage, acting through several mechanisms such as preventing DNA cleavage, competing with ATP for the same binding site [13,14,15] to prevent ATP hydrolysis, and interfering with DNA–topo II binding [16].

The design of dual-target or multitarget inhibitors is a promising approach to overcome cancer cell resistance and the side effects of topoisomerase II inhibitors while reducing the pharmacokinetic issues associated with combinatorial therapy. One of the proposed strategies is to target proteins that are structurally related to topo II, such as Hsp90, and kinases that have similar ATP-binding domains [17]. Alternatively, molecular hybridization of two pharmacophores resulting in dual-binding inhibitors, such as the daunorubicin-suberoylanilide hydroxamic acid (SAHA) hybrid that targets topo II and histone deacetylases (HDACs), also presents itself as a viable new strategy [18]. 

Intracellular targets as well as the polypharmacological profiles of the newly studied anticancer agents are often discovered retrospectively. In silico tools that analyze pharmacophoric similarity with drugs with known mechanisms of action are often very useful for this task [19]. Moreover, the use of molecular dynamics (MD) simulations in combination with pharmacophore modeling is a successful approach in the development of potent telomerase inhibitors as anticancer drugs [20]. 

In the last 10 years, there has been a resurgence of interest in phenotypic drug discovery (PDD) in both academic research and the pharmaceutical industry [21]. PDD screening yields hit compounds with diverse mechanisms of action, but the further development of optimized compounds is complicated by additional variables such as cell permeability, potential polypharmacology, binding to transport proteins, and metabolic stability. Although there are successful examples of hit-to-lead optimization using ligand-based structure–activity relationships (SAR), lack of target knowledge is considered a major risk for clinical development and regulatory approval [22]. 

**Figure 1:**
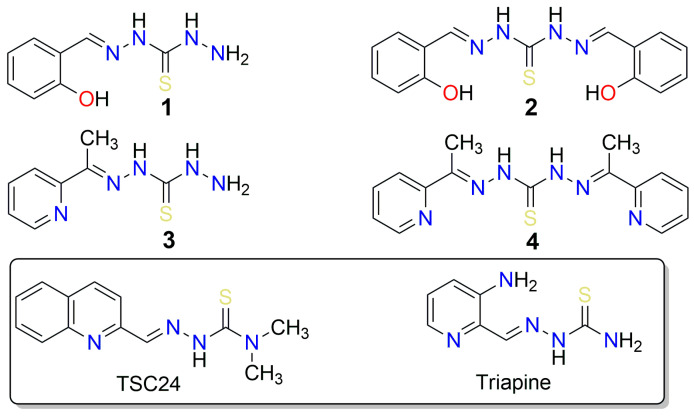
Structures of thiocarbohydrazones **1–4** investigated in this study, along with structurally similar thiosemicarbazones (triapine) that possess anticancer properties, and **TSC24,** which additionally inhibits the human DNA topoisomerase IIα.

Thiosemicarbazones (TSCs) are a well-known class of molecules with anticancer properties. Among them, the α-(N)-heterocyclic TSCs are of particular interest due to their ability to also chelate metal ions. These ligands bind strongly to Fe ions and inhibit metal-dependent enzymes such as ribonucleotide reductase, which is essential for DNA biosynthesis. The best-known example of this class of compounds is 3-aminopyridine-2-carboxaldehyde TSC (3-AP, triapine), a ribonucleotide reductase inhibitor that was tested in more than 30 Phase I and Phase II trials and is currently in Phase III clinical trials for radiotherapy in combination with cisplatin [23]. The inhibitory effect of triapine on topo II was investigated. Yalowich et. al. reported that triapine and some other TSCs did not induce cleavage of plasmid DNA or inhibit topo IIα decatenation [24]. On the other hand, Huang et. al. reported a series of α-(N)-heterocyclic TSC derivatives (Figure 1), such as compound **TSC24,** that act as catalytic inhibitors of topo IIα and bind to the ATPase domain where the ATP binding site is located [25].

Thiocarbohydrazones (TCHs) are higher homologs of TSCs with an additional N atom that can act as a metal-coordinating center. Compared to TSCs, the reports on the anticancer activity and mechanistic studies of TCHs are scarce. In a previous study, a series of mono- and bis-TCHs displayed a polypharmacological profile of anticancer activity with strong indications of a multitarget mechanism of action [26]. Moreover, salicylaldehyde monothiocarbohydrazone was reported to act as a copper ion ionophore and an antiproliferative agent against breast cancer and human prostate adenocarcinoma cell lines with low toxicity to normal human keratinocytes [27]. It was reported that the addition of Cu^2+^ increased the antiproliferative activity of salicylaldehyde mono-TCH. In addition, the complex of 1,5-bis(salicylidene)thiocarbohydrazide (compound **2**, Figure 1) and Cu^2+^ formed in situ efficiently cleaved DNA via oxidative and hydrolytic pathways, resulting in significant antiproliferative activity against HeLa and MCF-7 cancer cell lines [28]. However, no studies on the activity of TCHs on topo IIα have been reported so far. 

Here, we report the phenotypic screening of a small series of mono- and bis-TCHs (salicylaldehyde or 2-acetylpyridine) bearing an α-(N)-atom or an α-hydroxyl group as metal chelating centers and radical scavenging groups (Figure 1) against acute monocytic leukemia (THP-1), breast adenocarcinoma (MCF-7), and pancreatic adenocarcinoma (AsPC-1) cell lines. Encouraged by the observed activity of these compounds and their similarity to the TSC-based compound **TSC24,** a known catalytic topo IIα inhibitor [25], we then investigated their topo IIα inhibitory activity. For the most promising compound (**2**), we performed additional biochemical assays to further investigate its mechanism of action. Computational study of the binding properties of the active compounds **2** and **TSC24** in the ATP binding site of topo IIα, with molecular simulations, dynamic pharmacophore models, and MM/GBSA binding free energy calculations enabled a deeper insight into molecular recognition and provided information for further optimization. We also performed target fishing and molecular docking to outline other plausible targets and evaluated TCH’s drug-like properties.

## 2. Results and Discussion

### 2.1. Phenotypic Screening of Thiocarbohydrazones on Human Cancer Cell Lines

First, we tested the proapoptotic effect of thiocarbohydrazones **1–4** on leukemia (THP-1), breast cancer (MCF-7), and prostate adenocarcinoma (AsPC-1) cancer cell lines. Apoptosis is the main type of cell death in THP-1 and MCF-7, whereas AsPC-1 proved to be a highly resistant cancer stem cell, with its cell death events being exclusively necrotic [29,30]. The obtained results shown in Table S1 demonstrate that compound **4** induced cell death in three cell lines and suggest a higher efficacy of bis-TCH derivatives compared to mono-TCHs. 

**Figure 2:**
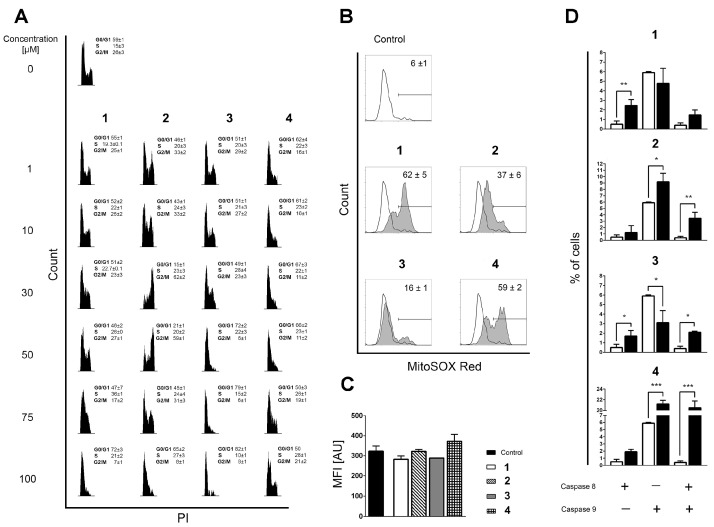
(**A**) Distribution of THP-1 cells within phases of mitotic division assessed on the remaining cells after Annexin V/PI readouts. Incidences of cells in phases G0/G1, S, and G2/M were determined according to the distribution of cells in nontreated populations. All results are expressed as mean ± SD of two replicates from independent experiments. (**B**) Mitochondrial superoxide (O_2_^●−^) generation in nontreated and cells treated with the investigated compounds, determined after 6 h of incubation by using MitoSOX Red staining. Results represent mean ± SD percentage of cells positive for mitochondrial O_2_^●−^ production in three replicates from independent experiments. (**C**) Mean fluorescent intensity (MFI) computed for O_2_^●−^-positive subpopulations, expressed in arbitrary units (AU). Results are presented as the mean ± SD of three replicates from independent experiments. The Kruskal–Wallis test showed no statistical difference in mitochondrial O_2_^●−^ accumulation between the analyzed samples. (**D**) Percentages of cells positive for activated caspase-8, caspase-9, or both caspases acquired in nontreated samples (open bars) or in samples treated for 6 h with the investigated compounds (closed bars). Bars represent the mean ± SD of three replicates from independent experiments. Statistical evaluation was performed using an unpaired *t*-test with Welch’s correction comparing treated to non-treated populations. Significant differences are marked with *, **, and ***.

The apoptotic response was concentration-dependent for all compounds except for compound **2**, 1,5-bis(salicylidene)thiocarbohydrazide (Figure S1), for which a non-standard biphasic curve with two exponential phases and a middle plateau situated between 10 and 50 µM was observed (Figure S2). These changes strongly correlate with the distribution of THP-1 cells within the phases of mitotic division (Figure 2A). The suspended increase in the percentage of apoptotic cells detected at 10 and 50 µM of compound **2** coincides with the cell cycle arrest at the G2 checkpoint. A burst in the incidence of cells in the advanced phases of apoptosis at 75 µM of compound **2** was accompanied by a decreased frequency of cells at the G2/M phase and an apparently restored G1 phase, whereas compound **2** at 100 µM stimulated arrest at the G1-to-S transition point. The described fluctuations in apoptotic response and cell cycle distribution imply that the DNA repairing machinery is initiated and operates in THP-1 cells treated with compound **2** within 10–50 µM concentration range, which strongly indicates that compound **2** interferes with chromosomal replication. At this point, it remains unclear whether compound **2** at concentrations greater than 50 µM affects the THP-1 cells via the same mechanism, or if another one emerges.

Although compounds **1**, **3,** and **4** also arrested the THP-1 cells at the G0/G1 phase or at the G1-to-S checkpoint, none of these changes provide an indication that DNA repair is ongoing at any of the concentrations tested (Figure 2A). DNA replication is a very complex process that may be interfered with in various ways [31,32]. Therefore, it was interesting to determine whether the currently investigated compounds stimulate the production of reactive oxygen species (ROS) that may damage DNA integrity and compromise its replication [33]. Therefore, we assessed the impact of compounds **1–4** on the mitochondrial superoxide anion (O_2_^●−^) production in mitochondria, which is the cellular organelle known as the key source of ROS generation. The compounds display quite different pro-oxidant activity regarding the percentage of cells positive for O_2_^●−^ (Figure 2B), but the values of mean fluorescence intensity (MFI) in all treated samples are on the level of non-treated controls (Figure 2C). The MFI results indicate that O_2_^●−^ does not accumulate but is most likely rapidly neutralized by the mitochondrial superoxide dismutase [34], implying that DNA damage by ROS is unlikely to be the reason why DNA repair machinery was launched after the treatment of THP-1 cells with compound **2**. 

### 2.2. Similarity-Based Quest for Possible Targets: Human DNA Topoisomerase IIα 

In search of the possible molecular targets that could rationalize the observations obtained in the phenotypic screening, we came across a structurally similar thiosemicarbazone derivative called compound **TSC24** (Figure 1). To quantify its similarity with our series, we calculated the Tanimoto maximum common structure similarity (MCS) index for compounds **2** and **3** with **TSC24** and obtained values of 0.60 and 0.38, respectively [35,36]. This compound acted as a human topo IIα catalytic inhibitor and potentially targeted the ATP binding site on the enzyme’s ATPase domain. To test whether the thiocarbohydrazones target the same enzyme, we first screened compounds **1–4** and the etoposide, a known topoisomerase poison, at a concentration of 50 µM with HTS topo IIα relaxation assay [37]. The assay was performed as previously described [15]. The selected screening concentration corresponded to the concentration that induced the cell cycle arrest at the G2 phase in THP-1 cells treated with compound **2** (Figure 2A). The percentage of topo IIα inhibition for etoposide was in line with the literature [13], and the results (Table S2) showed first indications that the α-hydroxyphenyl-thiocarbohydrazones (TCHs) can inhibit topo IIα. The most promising compound was 1,5-bis(salicylidene)thiocarbohydrazide (compound **2**) with a higher percentage of inhibition than etoposide (83% vs. 70%) at 50 µM. This highlights that compound **2** may be a potential inhibitor of topo IIα, and it allows speculation on the underlying cause of the arrest of THP-1 cells at the G2 checkpoint after treatment with 50 µM of compound **2** (Figure 2A). Moreover, the activation of the intrinsic apoptotic pathway for bis-TCH analog compound **2** suggests that the damaged cellular homeostasis is responsible for its pro-apoptotic activity (Figure 2D) [38,39]. 

**Figure 3:**
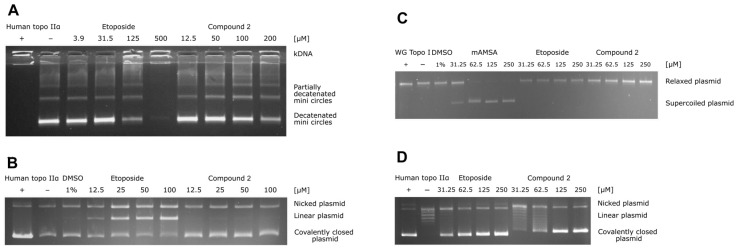
(**A**) Results of the human topo IIα-mediated decatenation assay. The assay was performed for four different concentrations of compound **2** (12.5, 50, 100, and 200 μM) and for etoposide as a positive control (3.9, 31.5, 125, and 500 μM). (**B**) Human topo IIα-mediated cleavage assay. The assay was performed for four different concentrations of compound **2** (12.5, 25, 50, and 100 μM), and etoposide was used as a positive control (12.5, 25, 50, and 100 μM). (**C**) Unwinding assay using supercoiled substrate. The assay was performed for four different concentrations of compound **2** and etoposide (31.5, 62.5, 125, and 250 μM) and of the positive control intercalator mAMSA (31.5, 62.5, 125, and 250 μM). (**D**) Results of the topoisomerase IIα-mediated relaxation assay. The assay was performed for four different concentrations of compound **2** (31.5, 62.5, 125, and 250 μM) and etoposide as positive control (31.5, 62.5, 125, and 250 μM).

To study the mechanism of topo IIα inhibition in detail, additional assays are required, as topo II operates as a complex molecular machine [40]. Thus, we focused on the most promising thiocarbohydrazone, compound **2**, from the initial HTS assay and performed a topo IIα-mediated decatenation assay together with etoposide as a reference molecule. The results of this assay allow direct observation of the inhibition process on the gel. Analysis of the data obtained for etoposide showed that the first traces of decatenated kinetoplast DNA (kDNA) appeared at 125 µM and became more intense at 31.5 µM, which is consistent with the literature (Figure 3A). Furthermore, compound **2** inhibited the decatenation of kDNA in a concentration-dependent manner, with 56% inhibition occurring at 50 µM (Figure 3A) (see also Figure S3A and Table S3 for more details). 

To distinguish the catalytic inhibitors from the topoisomerase poisons, we performed a topo IIα-mediated cleavage assay. The topoisomerase II poisons act by stabilizing the short-lived DNA-enzyme cleavage complex, which prevents the relegation of the plasmid and leads to the accumulation of the linear, cleaved plasmid. The results shown in Figure 3B (and in Figure S3B and Table S4) confirm that etoposide gradually increases the amount of linear plasmid. In the case of compound **2**, there was no significant increase in linear plasmid at any concentration tested, indicating that compound **2** acts as a catalytic inhibitor of topo IIα. 

In addition, we examined the activity of compound **2** as a DNA intercalator using the unwinding assay with mAMSA as a positive control. The results in Figure 3C and Figure S3C show that the intercalation of mAMSA was completed at 62.5 µM, whereas no unwinding was observed even at 250 µM of compound **2** with supercoiled or relaxed substrate. Therefore, compound **2** is not a DNA intercalator. To obtain further direct evidence of topo IIα inhibition, we also performed a topo IIα-mediated relaxation assay on the gel. The results shown in Figure 3D (and in Figure S3D and Table S5) independently confirmed the results of the decatenation assay, i.e., that compound **2** inhibits topo II-mediated DNA topology changes in a concentration-dependent manner (e.g., 61% inhibition at 62.5 µM). Taken together, the performed biochemical assays strongly imply that compound **2** acts as a catalytic inhibitor of human topo IIα. 

### 2.3. Thiosemicarbazide and Thiocarbohydrazone Binding to Topo IIα ATPase Domain Investigated with Molecular Simulations

**Figure 4:**
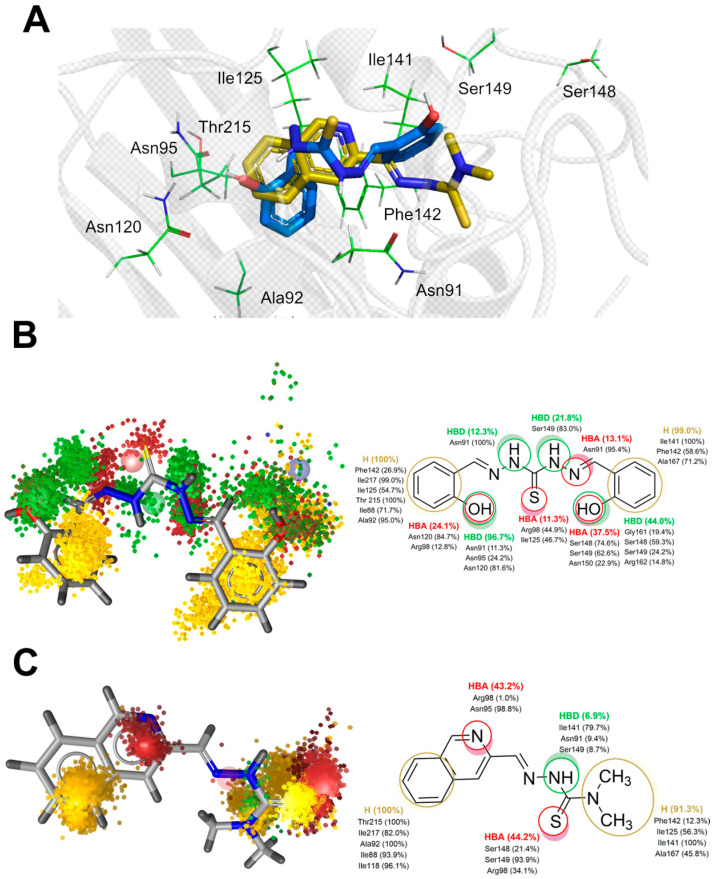
(**A**) Representative binding poses of compounds **2** and **TSC24** within the ATPase domain of human topo IIα obtained via molecular docking (PDB:1ZXM); dynophore models represented as superfeature clouds (left) and the contribution of individual amino acid residues to each interaction (right) for compounds **2** (**B**) and **TSC24** (**C**).

The structural resemblance between thiosemicarbazone **TSC24** and thiocarbohydrazone **2** further implies that both compounds could target the ATP binding site located on the topo IIα ATPase domain. Currently, there is no available crystal structure of human topoisomerase IIα in complex with a catalytic inhibitor bound to the ATP binding site; thus, the binding mode of **TSC24** was proposed with molecular docking using the available structure of the ATPase domain of human topo IIα, as in the original study [25]. The quinoline moiety of **TSC24** was placed deep in the binding pocket, forming mainly hydrophobic interactions with Ile125, Ile141, and Phe142, as well as with Asn95 and Asn120 residues located within the adenine sub-pocket (Figure 4A). This binding mode differs from the previously published mode [25], wherein the α-N atom of the quinoline moiety was reported to interact with the hydroxyl group of Ser149. This placed the **TSC24** ligand in the middle of the adenine binding pocket near the sugar-binding region. This difference in binding poses likely originates from the inclusion of a Mg^2+^ ion in the definition of the active site [25]. As Mg^2+^ generally binds to the enzymes in a complex with ATP [41], we omitted this ion from the active site in our docking experiments, as such a binding site topology could be considered more realistic [13,14,15,40]. 

We also docked thiocarbohydrazone **2**, and one of its phenolic moieties was positioned within the adenine part of the ATP pocket, where it interacted with residues Asn120 and Asn95. The hydrazide NH atoms were located near Asn91, a part of the phosphate-binding region of the ATPase domain. Both aromatic rings of compound **2** were oriented towards the hydrophobic pocket and comprised residues Ile125, Ile141, and Phe142 (Figure 4A). 

Starting from the docking poses of ligands **TSC24** and **2** in the ATP binding site, we then performed 200 ns of unrestrained MD simulations of a fully solvated system. For compound **2**, we observed in the performed replica 1 (R1) simulation that the interactions with residues defining the adenine subpocket (e.g., Asn120 and Asn95) as well as the hydrophobic subpocket (Ile125, Ile141, and Phe142) were mostly conserved during the entire trajectory. On the other hand, the thiocarbohydrazide linker and the second phenolic group adopted two orientations in the binding site, which we labeled as CF1 and CF2 (Figure S4). To better capture the dynamics of these interactions, we performed two additional simulations termed R2 and R3, each with a length of 200 ns that started from the different initial structures generated by prolonging the NPT equilibration stage. The results indicate that the CF1 and CF2 orientations were exchanged throughout the entire trajectory. The root mean square deviations (RMSD) of the ligand **2** for three replicas of the MD simulation ranged from 1.28 Å for R1 to 1.75 Å for R3 (Figure S5A and Table S6). The bound conformation of the ligand **TSC24** displayed less conformational variation within the ATP binding site during the simulations. Lower average RMSD values of the ligand, between 0.92 Å for the first simulation (R1) and 0.95 Å for the second replica (R2), suggested general conformational confinement to the docking mode (Figure S5B and Table S7). Because the **TSC24** binding conformation was strongly preserved and stable in both replicas R1 and R2, we did not perform a third simulation. 

To complement the geometric analysis of the binding modes of thiocarbohydrazone **2** and the compound **TSC24**, we also calculated the dynamical pharmacophores (dynophores) [42] for all performed simulations. With this approach, one can more effectively investigate the hydrophobic interactions as well as H-bonds between the ligand and the binding site. The dynophore models for the R2 simulation of compound **2** and R2 of **TSC24** are shown in Figure 4B,C. The remaining models and their corresponding animations can be found in the Supplementary Information (Figures S6–S8 and Videos S1–S5).

Dynophores pinpointed the favorable hydrophobic interactions between the first phenolic moiety of compound **2** and residues Ile125, Ile217, Ile88, Phe142, Thr215, and Ala92, which contribute to the stability within the ATP binding site. Furthermore, the hydrogen bonds of the phenolic OH of compound **2** with Asn91, Asn95, Arg98, and particularly Asn120 were conserved during the MD trajectories, providing the additional stabilization of the ligand in the binding pocket. The conformational mobility of the thiocarbohydrazide linker and the second phenolic group of compound **2** observed in the simulation, oscillating between the orientations CF1 and CF2, resulted in a scattered pattern of pharmacophore super-features around the amino acids responsible for sugar and phosphate binding of the ATP molecule. More precisely, the thiocarbohydrazide linker established hydrogen bond donor (HBD) interactions with Asn91, Ser149, Asn150, and Arg162 in approximately 30% of the simulation time. The hydrogen bond acceptor (HBA) interactions of the thioketo sulfur atom with Arg98 and Ile125 were observed in 11% of the simulation time. The second phenolic group, located in the outer part of the binding site, was bound to Ser148, Ser149 and Asn150 via HBA interactions. This part of the molecule was also stabilized by hydrophobic interactions with Ile141, Phe142, and Ala167 (Figure 4B). Conformational analysis of the second replica of the MD simulation (Supplementary Video S2) shows that ligand **2** preferred the bent conformation (CF1). The driving forces for this conformational change of the ligand to its linear conformation CF2 appear to be interactions with residues of the ATP-sugar binding region as well as the ATP-binding loop, i.e., residues Ser148, Ser149, Asn150, Arg162, Asn163, Gly164, and Ala167. 

Compared to a larger conformational space that bound compound **2** exhibits during the simulations, the ligand **TSC24** remains predominantly in a single conformation. The quinoline moiety of the ligand is located in the adenine binding pocket of the binding site and forms hydrophobic interactions with Ile88, Ala92, Ile118, Thr215, and Ile217 and HBA interactions with Asn95 and Arg98. Two interactions that stabilize compound **2** within the adenine subpocket were not found for quinoline ring of **TSC24**; hydrogen bond interaction with Asn120 and hydrophobic interactions with the hydrophobic pocket comprised Ile125, Ile141, and Phe142. Two methyl groups of the thiosemicarbazone moiety established stable hydrophobic interactions with this pocket and with Ala167. This ligand was additionally stabilized by HBD interactions with the main chain of Ile141 and HBA bonds with Arg98, Ser148, and Ser149 (Figure 4C). 

Overall, the bound conformation of **TSC24** exhibited a more conformationally restricted and stable pattern of interactions than compound **2**, where determined interactions were more dispersed. Decatenation and relaxation experiments demonstrated that at the 25 μM concentration of **TSC24**, topo IIα was completely inhibited [25], and it thus exhibited better inhibition properties than compound **2**. The obtained simulation data imply that optimization of the flexible thiocarbohydrazide linker of this lead compound by either structure rigidification and/or more efficient exploitation of the interactions revealed from the dynophore models could pave the way to more potent and selective topo IIα-acting compounds. 

To complement the geometry-based information with energetic data, we performed Molecular Mechanics/Generalized Born Surface Area (MM/GBSA) free energy calculations. This approach does not consider explicit water molecules that might be involved in hydrogen bonding between the protein and the ligand. Instead, the explicit solvent is removed and replaced with an implicit continuum solvent to significantly speed up computation time. Interestingly, this calculation suggested that compound **2** binds more strongly to the ATPase domain compared to **TSC24** (ΔG = −34.92 ± 6.14 vs. −29.71 ± 3.78 kcal/mol, respectively (Figure S9)). Although the average binding affinity of compound **2** was higher overall for topo IIα, it was also associated with a significantly higher standard deviation of ΔG compared with conformationally restrained **TSC24**, consistent with the more stable binding position observed for **TSC24**. 

To evaluate the importance of the observed interactions, we analyzed the energy contributions of the individual residues that were shown by the dynophore models to stabilize the ligands in a particular conformation (Table 1). The results show that the hydrogen bond interactions with Asn91, Asn95, and Asn120 contributed most strongly to the stability of a phenolic moiety of compound **2** bound in the adenine binding pocket. Hydrophobic interactions with Ile125, Ile141, Phe142 also stabilized compound **2** in CF1 and CF2. The main differences between the two stable conformations CF1 and CF2 are the smaller contribution of Phe142 in the linear conformation CF2 as the second phenol ring shifts away, weaker interactions of CF2 with the adenine-binding part, and stronger interactions with residues Arg98, Asn163, and Gly164 of the ATP-binding loop. 

The reference ligand **TSC24** forms hydrophobic interactions with Ala92, Ile217, Ile125, Ile141, and Phe142 of the adenine binding region (Table 1). MM/GBSA analysis of the two selected 20 ns timeframes of the trajectory revealed that Asn95 and Arg98 are not important for molecular recognition between **TSC24** and the topo IIα catalytic domain in contrast to the dynophore analysis (Figure 4). The interactions of the aliphatic part of **TSC24** with Ser148 and Gly164 of the phosphate binding part of the ATPase binding site were also suggested to be important for the stability of the protein/ligand complex by analyzing the MM/GBSA calculations.

**Figure 5:**
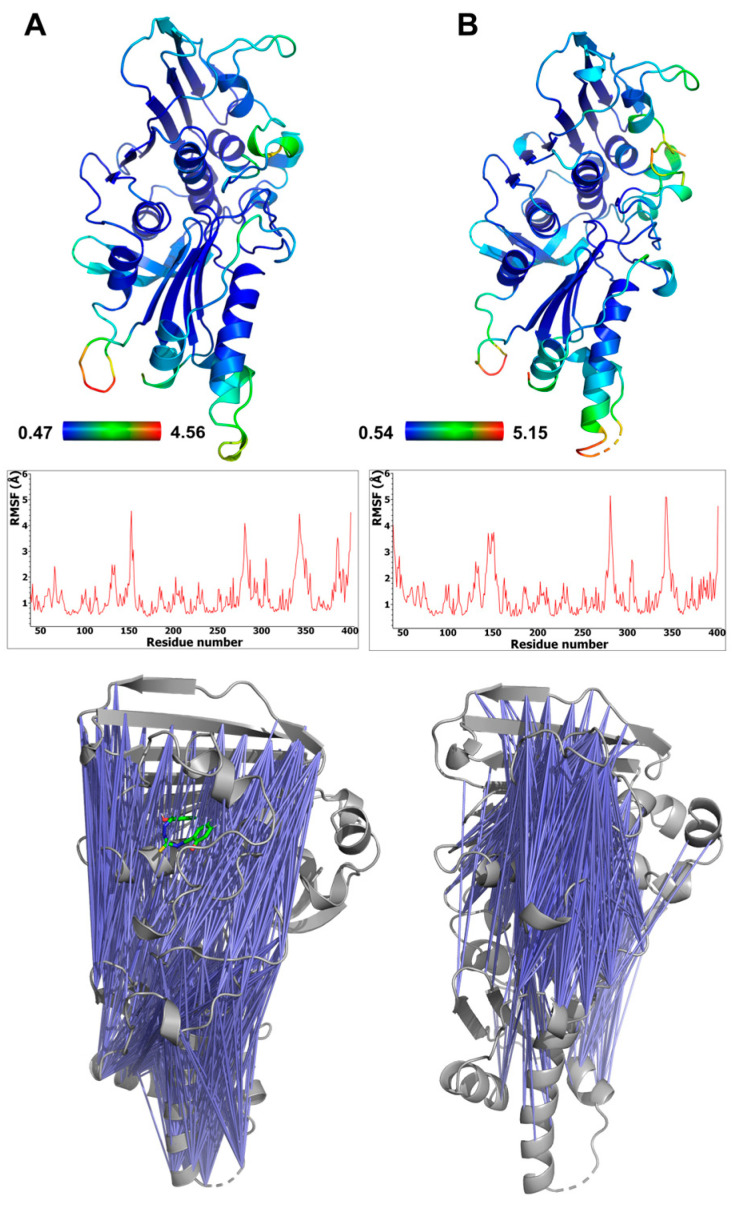
Flexibility of the ATPase domain of human topo IIα, depicted as a B-factor representation colored by RMSF values (top) and also shown as RMSF graphs s (middle) for complexes with compound **2** (**A**) and **TSC24** (**B**). The bottom panel shows pairs of residues of topo IIα ATPase domain displaying anticorrelation movements (correlation coefficients between −0.4 and −0.6) (left), topo IIα complex with compound **2**, and (right) topo IIα complex with compound **TSC24**.

The conformational behavior of the ATPase domain of topo IIα with both bound compounds was also investigated. The average RMSD of the protein backbone for the topo IIα-**TSC24** complex was between 1.66 and 1.70 Å, whereas for the three replicas of topo IIα with bound compound **2**, the averages were 2.01, 2.15, and 2.49 Å (as shown in Tables S6 and S7). The temporal RMSD plots show that complexes reached equilibrium after about 20–30 ns of simulation. To further pinpoint the main source of flexibility, we performed root-mean-square fluctuation (RMSF) calculations [43], where the most profound fluctuations were associated with the movement of residues in the transducer domain of topo IIα, comprising residues 265–405 (Figure 5A), which is similar to observations in our previous studies [5]. The protein flexibility induced by the binding of the two catalytic inhibitors is similar, and two conformations of compound **2** revealed by MD (Figure S4) did not influence protein flexibility considerably (Table S6 ). The adenine and sugar-binding moiety of the ATP binding site were essentially unchanged during the simulations, whereas some structural fluctuations were observed in the ATP binding loop and the hydrophobic pocket above the ATP binding site. 

The calculated cross-correlation matrices of the protein residues revealed significant anticorrelation movements of the ATP binding site residues located on the GHKL domain and the residues of the transducer domain. These pairs of residues in complex with bound **TSC24** displayed more pronounced and focused anticorrelations around the ATP binding site compared to complexes with compound **2** (Figure 5), which is in line with its superior topo IIα inhibition [25]. The binding of both compounds results in enhanced and focused movement compared to the apo structure of topo IIα we simulated in our previous study, which showed considerably more randomly distributed anticorrelation movements [44]. This indicated that the ligands can mimic some of the interactions of the native ATP ligand, which was also able to enhance the focused movement compared to the apo structure [44].

### 2.4. In Silico Target Fishing of Thiocarbohydrazones and Assessment of Drug-Likeness 

Phenotypic screening (Figure 2A and Figure S2) indicated that the cytotoxic activity of compound **2** is probably associated with the activity on multiple targets. In this study, we evaluated that compound **2** is a catalytic inhibitor of human DNA topoisomerase IIα. To gain further insights and deconvolute the results of the phenotypic screening, we searched for additional potential macromolecular targets using the PharmMapper web server [45]. This program analyzes the pharmacophoric similarity between the multiple conformations of the input structure and several thousand pharmacophore models mapping the active sites of different protein targets. The results indicated that glutathione S-transferase P, carbonic anhydrase 12, and cell division protein kinase 2 were the three best-matched proteins associated with cancer (Table S8). 

Next, we docked compound **2** into the active site of these three proteins, relaxed the obtained complexes using a short MD simulation to remove steric hindrances, and rescored binding poses using the ChemPLP, PLP, and PLP95 scoring functions [46]. The docking scores highlighted cell division protein kinase 2 (CDPK2) as a promising target (Table S9). The binding mode of compound **2** in the active site of CDPK2 shows that two phenolic moieties establish a rich pattern of hydrophobic, HBD, and HBA interactions within the binding pocket (Figure S10). These data provide a starting point for future studies to further evaluate the mode of action of these compounds. 

According to the Biopharmaceutics Classification System (BCS) and the estimated ADMET properties (Table S10), compound **2** belongs to Class I, i.e., compounds with high permeability and high solubility. It follows Lipinski’s rule of five [47] and shows promising bioavailability; thus, it is likely to be well absorbed upon oral administration. Moreover, compound **2** has low blood–brain barrier (BBB) permeability and is not a substrate for P-glycoprotein. In terms of potential toxicity, compound **2** is predicted as non-hepatotoxic but could inhibit the hERG II ion channel. In addition, the hydrazone phenol moiety of compound **2** is recognized as a potential pan-assay interference structure (PAINS), which urges caution in further development. Nevertheless, some drugs used in the clinic such as the anticancer agent mitoguazone, the antibiotic agent nifuroxazide and the antihypertensive agent dihydralazine possess a hydrazone moiety [48]. Compound **2** thus possesses lead-like properties; however, future modifications should aim to reduce potential cardiotoxicity and carefully validate the PAINS potential or, preferably, eliminate it completely.

## 3. Materials and Methods

### 3.1. Chemistry

The synthesis, characterization, and isomerism of thiocarbohydrazones **1–4** were reported in our previous paper [49]. 

### 3.2. Biology

#### 3.2.1. Cell Culture and Preparation of Solutions

Human acute monocytic leukemia cell line (THP-1, ATCC^®^ TIB-202) was maintained in RPMI-1640 (Life Technologies, Paisley, UK, Cat. No. 11875-093), supplemented with 10% (*v*/*v*) heat inactivated fetal bovine serum (FBS, Life Technologies, Paisley, UK, Cat No 10270-106) and 1% (*v*/*v*) penicillin-streptomycin (10,000 units/mL and 10,000 µg/mL, Life Technologies, Paisley, UK, Cat No 15140-122). Cells were kept at 37 °C in a humidified atmosphere containing 5% (*v*/*v*) CO_2_ during their exponential growing phase and during incubation with investigated compounds. 

Investigated compounds **1–4** were dissolved in DMSO to the stock concentration of 20 mM. Further dilutions to the experimental concentrations applied on the cells were done with RPMI-1640 or DMEM media immediately before each experiment; thus, the final concentration of DMSO on cells treated with the highest applied concentration of an investigated compound was 0.5% (*v*/*v*).

#### 3.2.2. Annexin V and Propidium Iodide Staining

Cells were seeded in 96 flat-bottom well plates (Corning^®^ Costar^®^, Cat. No. CLS3596) in a volume of 0.1 mL at a density of 10,000 per well. Investigated compounds **1–4** were added in a range of six concentrations 24 h after cell seeding. As controls, non-treated cells, cells treated with 0.5% DMSO, and cells treated with Celastrol (Enzo Life Sciences, Lausen, Switzerland, Cat. No. ALX-350-332-M025) at 50 µM concentration were used. After 24 h of treatment, Annexin V-FITC (Immuno Tools, Friesoythe, Germany, Cat No 31490013) and propidium iodide (PI, Miltenyl Biotec Inc., Auburn, AB, USA, Cat No 130-093-233) were added to wells in a volume of 3 µL each. Plates were analyzed after 30 min of incubation in the dark with a Guava^®^ easyCyte 12HT Benchtop flow microcapillary cytometer (Millipore, Merck, Darmstadt, Germany) using the dedicated InCyte^TM^ 3.1 software package. Cells were classified according to Annexin V-FITC (green fluorescence) and PI (red fluorescence) labeling on viable cells (double negative), pre-apoptotic cells (Annexin V-FITC single-stained cells), necrotic cells (PI single-stained cells), and cells in advanced phases of apoptosis (double-stained cells). 

#### 3.2.3. Concentration–Response Curve Plotting

Percentages of Annexin V single-stained and double-stained cells were summarized for each concentration of investigated compound and plotted against corresponding concentrations. Concentration–response curves were drawn using the sigmoidal asymmetric five-parameter logistic equation, or the biphasic model, for the hill-shaped curve in GraphPad Prism 6 software (GraphPad Software, Inc., Boston, MA, United States). 

#### 3.2.4. Cell Cycle Analysis

The distribution of cells within phases of mitotic division was evaluated on the remaining cells after Annexin V/PI analysis, which, right after the readout was finished, were fixed in ethanol overnight at 4 °C. Before reading, plates were centrifuged on 450× *g* for 10 min, ethanol was discarded, and PBS was added in a volume of 100 µL per well. Cells were stained with 50 µL of FxCycle^TM^ PI/RNAse Staining solution (Molecular Probes, Eugene, OR, United States; Thermo Fisher Scientific, Waltham, MA, United States, Cat. No. F10797) and incubated at 37 °C for 30 min in the dark. Plates were analyzed with a Guava^®^ easyCyte 12HT Benchtop flow microcapillary cytometer using the dedicated InCyte^TM^ 3.1 software package.

#### 3.2.5. Caspase-8 and Caspase-9 Activities

Cells were treated with investigated compounds at 50 µM concentration for 6 h; afterwards, the activity of caspase-8 and caspase-9 were assayed by means of Guava Caspase 9 SR and Caspase 8 FAM kits (EMD Millipore, Merck, Darmstadt, Germany, Cat. No. 4500-0640) by following the manufacturer’s instructions. Cells were analyzed with a Guava^®^ easyCyte 12HT Benchtop flow microcapillary cytometer using the dedicated InCyte^TM^ 3.1 software package. Acquired data cells were discriminated according to their expression of caspase-8 (Grn-B fluorescence) and caspase-9 (Yel-B fluorescence).

#### 3.2.6. Generation of Radical Oxygen Species in Mitochondria

Cells were treated over 6 h with investigated compounds in a concentration of 50 µM; afterwards, they were stained with MitoSox Red (Molecular Probes, Cat. No. M36008) according to the manufacturer’s recommendations. Analysis was performed with a Guava^®^ easyCyte 12HT Benchtop flow microcapillary cytometer using the dedicated InCyte^TM^ 3.1 software package. The generation of O_2_^●−^ was evaluated by means of two parameters: percentage of O_2_^●−^-generating cells, and mean fluorescence intensity (MFI) expressed in arbitrary units (AU). The MFI was computed for the O_2_^●−^-positive subpopulation, and it indicated the average quantity of O_2_^●−^ per cell.

#### 3.2.7. Human DNA Topoisomerase IIα-Mediated Decatenation Assay

This topo IIα assay, along with additional assays described in Section 2.2, were performed in collaboration with Inspiralis (Norwich, UK). One U of topo II was incubated with 200 ng kDNA in a 30 μL reaction at 37 °C for 30 min under the following conditions: 50 mM Tris HCl (pH 7.5), 125 mM NaCl, 10 mM MgCl_2_, 5 mM DTT, 0.5 mM EDTA, 0.1 mg/mL bovine serum albumin (BSA), and 1 mM ATP. The reaction was then stopped by the addition of 30 μL chloroform/isoamyl alcohol (26:1) and 30 μL Stop Dye (40% sucrose (*w*/*v*), 100 mM Tris. HCl (pH 7.5), 10 mM EDTA, 0.5 μg/mL bromophenol blue) before being loaded on a 1% TAE gel run at 85 V for 90 min. 

Bands were visualized via ethidium bromide staining for 15 min and destained for 10 min. Gels were scanned using documentation equipment (GeneGenius, Syngene, Cambridge, UK), and inhibition levels were calculated from the band data obtained with the gel scanning software (GeneTools, Syngene, Cambridge, UK). Assays were performed for active compound **2** at concentrations of 12.5, 50, 100, and 200 μM and for etoposide standard at concentrations of 3.9, 31.5, 125, and 500 μM.

#### 3.2.8. Human DNA Topoisomerase IIα-Mediated Relaxation Assay

The activity of the enzyme was determined prior to the testing of the compounds, and 1 U was defined as the amount of enzyme required to fully relax the substrate. Compound **2** was tested at 31.25 μM, 62.5 μM, 125 μM, and 250 μM, and these volumes were added to the reaction before the addition of the enzyme. Final DMSO concentration in the assays was 1% (*v*/*v*). One U of human topo IIα was incubated with 500 ng supercoiled pBR322 in a 30 μL reaction at 37 °C for 30 min under the following conditions: 50 mM Tris HCl (pH 7.5), 125 mM NaCl, 10 mM MgCl_2_, 5 mM DTT, 0.5 mM EDTA, 0.1 mg/mL bovine serum albumin (BSA), and 1 mM ATP. Each reaction was stopped by the addition of 30 μL chloroform/isoamyl alcohol (24:1) and 30 μL Stop Dye before being loaded on a 1.0% TAE gel run at 90 V for 90 min. Bands were visualized via ethidium staining for 10 min, destained for 10 min in water, analyzed by using gel documentation equipment (Syngene, Cambridge, UK), and quantified using Syngene Gene Tools software. Raw gel data (fluorescent band volumes) collected from Syngene, GeneTools gel analysis software were calculated as a percentage of the 100% control (the fully supercoiled DNA band) and converted to percent inhibition.

#### 3.2.9. Human DNA Topoisomerase IIα Cleavage Assay

The activity of the human topoisomerase IIα was determined prior to testing of the compounds, and 1 unit (U) was defined as the amount of enzyme required to reach the maximum cleavage of the substrate. The final DMSO concentration in all reactions was 1% (*v*/*v*). The compound was serially diluted in 100% DMSO and added to the reaction before the addition of the enzyme. The control compound for all assays was etoposide. One U of the human topo IIα was incubated with 0.5 μg supercoiled plasmid DNA (pBR322) in a 30 μL reaction at 37 °C for 30 min under the following conditions: 20 mM Tris HCl (pH 7.5), 200 mM NaCl, 0.25 mM EDTA, and 5% glycerol. The reaction was then incubated for a further 30 min with 0.2% SDS and 0.5 μg/μL proteinase K. The reaction was then stopped by the addition of 30 μL chloroform/isoamyl alcohol (26:1) and 30 μL Stop Dye (40% sucrose (*w*/*v*), 100 mM Tris HCl (pH 7.5), 10 mM EDTA, 0.5 μg/mL bromophenol blue) before being loaded on a 1% TAE gel run at 80 V for 2 h. Assays were performed for compound **2** at concentrations of 12.5, 25, 50, and 100 μM and for etoposide (control) at concentrations of 12.5, 25, 50 and 100 μM. Bands were visualized as scanned and as described in the decatenation assay.

#### 3.2.10. Wheatgerm Topo I Unwinding ASSAY

A volume of 1 U of wheatgerm topo I was incubated with 0.5 μg supercoiled or relaxed plasmid DNA (pBR322) in a 30 μL reaction at 37 °C for 30 min under the following conditions: 50 mM Tris HCl (pH 7.9), 50 mM NaCl, 1.0 mM EDTA, 1.0 mM DTT, and 20% glycerol. Each reaction was stopped, and the compounds were removed, prior to the running of the gels by the addition of 50 μL butanol and 30 μL of water. The samples were vortexed, and the aqueous layer was removed before the addition of 30 μL chloroform/iso-amyl alcohol (24:1) and 30 μL Stop Dye. These were then loaded on a 1.0% TAE gel run at 90 V for 2 h. Bands were visualized via ethidium bromide staining for 15 min and destaining for 10 min. Gels were scanned using documentation equipment (GeneGenius, Syngene, Cambridge, UK). The assay was performed at four different concentrations of compound **2** (31.5, 62.5, 125, and 250 μM) and of the positive control, intercalator mAMSA (31.5, 62.5, 125, and 250 μM).

### 3.3. Molecular Modeling

#### 3.3.1. Molecular Docking Calculations

The generated conformations of compounds **2** and **TSC24** were optimized with a MMFF94 force field [50], with the obtained conformations additionally optimized by using the semiempirical PM7 method [51] implemented in MOPAC2016 [52]. This approach generated structures with precise bond lengths and optimal conformational properties to provide a good starting geometry for the molecular docking. The active site of one protomer of human topo IIα ATPase domain (PDB code:1ZXM [53]) was defined as all residues up to 10 Å away from the co-crystallized ligand AMP-PNP. The ligand, water, and metal ions were removed, and the hydrogen atoms were added to resemble the protonation state of the protein at pH 7.4 as predicted by PROPKA [54]. The .pdbqt files of compound **2**, **TSC24**, and the receptor were prepared in Vega ZZ 3.2.0 [55,56]. AutoDockVina 1.1 [57] was used for docking. Exhaustiveness was set to 100, and 20 binding poses were stored. All other settings were kept at default values. 

#### 3.3.2. Molecular Dynamics Simulations

The best docking poses of compounds **2** and **TSC24** were taken as starting points for the protein–ligand complexes used for molecular dynamics simulations. We first determined the force field parameters for both ligands. The molecular geometry and electronic structure of compounds **2** and **TSC24** were first optimized at the Hartree–Fock level using the 6–31 G* basis set. The partial charges of the ligands were obtained by performing a Merz–Kollman population analysis. Quantum mechanical calculations were carried out in Gaussian 16, revision C.01 [58]. The RESP charges were then fitted using the Antechamber module of Amber18 [59]. The bond distances, bond angles, and dihedrals of the ligands were obtained from the optimized geometries using Antechamber. The force field parameters of the ligands were represented in the General Amber Force Field of second generation (gaff2). The partial atomic charges of the ligands **2** and **TSC24** and their atom types are provided in the Supplementary Materials, Tables S11 and S12. The monomer of human topo IIα ATPase domain (chain A, residues 39–345 and 350–405) comprised the simulated protein molecule.

Both complexes were solvated using a cubic box of TIP3P-type water molecules [60] with at least 10 Å distance between the edges of the box and the protein. Three chloride ions were added to neutralize the system, and the final system consisted of approximately 137,000 atoms. The Amber14SB force field was used to describe the protein [61], and gaff2 was used for the ligand atoms [62]. The energies of the solvated protein–ligand complexes were minimized by applying 10,000 steps of steepest descent minimization followed by 20,000 steps of conjugate gradient minimization. Next, we performed four NVT equilibration runs in which the system was gradually heated to 300 K using the Langevin thermostat. In each run, 10,000 steps with a time step of 2 fs were applied by gradually releasing the applied constraints on the protein from 100 kcal mol^−1^ Å^−2^ (first run) over 60 kcal mol^−1^ Å^−2^ (second) and 30 kcal mol^−1^ Å^−2^ (third run) to the fourth run, which had no restraints. Afterward, one run of 200 ps NPT equilibration with 2 fs time step was performed, applying 20 kcal mol^−1^ Å^−2^ force constants on the protein. The final run of NPT equilibration was done without applying any constraints. The pressure was maintained at 1 bar using the Berendsen thermostat [63]. 

The initial configurations of the three replicas of the MD simulation of the topo IIα-**2** complex were generated by varying the equilibration time of unconstrained NPT equilibration to 0.2, 0.6, and 1.0 ns. For the topo IIα-**TSC24** complex, 0.6 ns and 1.0 ns of the final NPT equilibration were taken for generating input structures of two replicas. The simulations were performed by applying periodic boundary conditions, with long-range electrostatics treated using the Particle Mesh Evald method [64] with the cut-off value of 10 Å. The lengths of all bonds involving hydrogen atoms were constrained using the SHAKE algorithm to achieve the time step of 2 fs [65]. Each of five MD replicas was 0.2 μs long with the production simulations done in the Amber18 pmemd.cuda program [66]. Molecular simulations were performed using the computational resources of the Azman high-performance computing (HPC) center at the National Institute of Chemistry in Slovenia.

#### 3.3.3. Analysis of the MD Trajectories

Molecular trajectories obtained during the production stage of the MD simulations were analyzed by determining the root-mean-square deviations (RMSD) of the protein backbone Cα and the ligand, which were calculated in the VMD software where the trajectories were also visualized [67]. The root-mean-square fluctuation (RMSF) values of the protein were calculated using the Cpptraj module of Amber 18 [59]. The initial structure of the protein–ligand complex was used as a reference frame for the RMSD and RMSF calculations. Dynamic pharmacophores were calculated using DynophoreApp software and using 400 equidistant frames for each simulated system [42]. 

The cross-correlation maps determining the extents of the pairwise residual correlations were calculated with the Bio3D package [68] and using its dccm function, which derived the covariation matrices and calculated the Pearson’s correlation coefficients ($C_{ij}$) on the Cα atom pairs, *i* and *j*. The anticorrelated movements of pairs of residues were visualized in PyMOL [69]. 

The binding free energy calculations of the protein–ligand complexes were performed using the Molecular Mechanics/Generalized Born Surface Area (MM/GBSA) method implemented in Amber Tools 20 [70,71]. Calculations were performed on 400 equidistant frames using the Generalized Born IGB method 5 and 0.15 M salt concentration. Moreover, we performed per-residue decomposition analysis to evaluate the contributions of individual amino acid residues to overall binding.

#### 3.3.4. Pharmacophore Similarity Search and ADME-Tox Predictions

The PharmMapper web server was used for the identification of other potential molecular targets for active compound **2** [45]. All protein targets from the v2010 database (7302 items) were matched against the database of pharmacophores generated for 300 conformers of compound **2** and ranked according to normalized Fit scores and Z-scores. The ADME-Tox properties of compound **2** were predicted using the Swiss-ADME [72] and pkCSM [73] online tools. The SMILES string of the compound was used as input.

## 4. Conclusions

Phenotypic screening of a small series of mono- and bis-substituted thiocarbohydrazones against three cancer cell lines revealed that 1,5-bis(salicylidene)thiocarbohydrazide **2** induces the apoptosis and activates the DNA repair machinery via an ROS-independent pathway. Structurally related thiosemicarbazones are known catalytic topo IIα inhibitors, and compound **2** exhibited a similar catalytic inhibition of action and did not act as a DNA intercalator. The molecular simulations of complexes of compounds **2** and **TSC24** with the ATPase domain of topo IIα, which were followed by dynamic pharmacophore and MM/GBSA analyses, revealed differences in the interaction patterns and conformational stability of the two ligands. Thiocarbohydrazide **2** was more flexible in the targeted ATP binding site compared with **TSC24**. Furthermore, the model of molecular recognition suggested that rigidifying the structure and optimizing the interactions with the sugar- and phosphate-binding portions of the topo IIα ATP site could result in more potent and selective derivatives. 

Human DNA topoisomerase IIα is a validated anticancer drug target, but rapidly evolving cancer resistance and severe side effects associated with the use of topoisomerase poisons such as etoposide and doxorubicin limit its applicability. The design and development of catalytic topo IIα inhibitors is a promising approach to overcome these issues. This study highlights 1,5-bis(salicylidene)thiocarbohydrazide as a potential novel lead compound with cytotoxic and topo II inhibition properties useful for chemotherapeutic anticancer drug discovery. 

## Figures and Tables

**Table 1:** The results of MM/GBSA binding free energy analysis for the selected timeframes in which ligands **2** and **TSC24** adopted a particular conformation. All residues with binding free energy contributions above 0.5 kcal/mol are listed and ordered by descending contributions.

	Replica, Timeframe	Conformation	Main Amino Acid Residues (Total Energy Decomposition ΔG in kcal/mol)
Compound **2**	R1, 40–60 ns	CF1, bent	Asn95 (−3.21), Asn91 (−2.97), Asn120 (−1.79), Phe142 (−1.43), Ile141 (−1.29), Ile 217 (−0.96), Thr215 (−0.92), Gly166 (−0.85), Ala167 (−0.85), Ile125 (−0.84)
	R1, 100–120 ns	CF2, linear	Asn91 (−1.85), Asn95 (−1.79), Ile141 (−1.78), Ile125 (−1.65), Asn120 (−1.47), Ile217 (−0.90), Arg98 (−0.83), Asn163 (−0.83), Phe142 (−0.79), Gly164 (−0.72), Thr215 (−0.67)
**TSC24**	40–60 ns	restrained	Ile141 (−2.17), Phe142 (−1.82), Asn91 (−1.06), Ala92 (−0.94), Ile125 (−0.94), Ser148 (−0.86), Thr215 (−0.85), Ile217 (−0.84), Gly164 (−0.78), Ile88 (−0.77)
	160–180 ns	restrained	Ala92 (−1.86), Ile217 (−1.79), Ile125 (−1.49), Gly164 (−1.09), Phe142 (−0.99), Ile141 (−0.98), Thr215 (−0.92), Asn91 (−0.80), Ile88 (−0.68), Asn120 (−0.67)

## Data Availability

Data are contained within the article and Supplementary Materials.

## Supplementary Materials

The following supporting information can be downloaded at: https://www.mdpi.com/article/10.3390/ph16030341/s1, Table S1: Anticancer activity of thiocarbohydrazones **1–4**; Figure S1: Types of cell death in THP-1 cells treated with investigated compounds, determined by means of Annexin V/PI dual staining assay after 24 h incubation; Figure S2: Concentration-response curve for compound **2** on THP-1 cells after 24 h treatment; Table S2: The results of HTS human topo IIα relaxation assay; Figure S3: The results of second run of human topo IIα decatenation assay, human topo IIα cleavage assay, unwinding assay, and topoisomerase IIα relaxation assay; Table S3: Results of decatenation assay (in duplicate) for thiocarbohydrazone **2** and etoposide at different concentrations represented as the % of the decatenated kDNA; Table S4: Percentage of linear DNA, determined in cleavage assay (in duplicate) for etoposide and thiocarbohydrazone **2** at four concentrations; Table S5: Results of relaxation assay (in duplicate) for thiocarbohydrazone **2** and etoposide at different concentrations represented as the % of topo IIα inhibition; Figure S4: Two conformations of compound **2** observed in three replicas of MD simulations; Figure S5: RMSD of the ligand heavy atoms for each replica of MD simulation of **2** (A) and **TSC24** (B) bound within the ATPase domain of human topo IIα; Table S6: Average RMSD of the ligand **2** and topo IIα protein for the three replicas of MD simulation; Table S7: Average RMSD of the **TSC24** ligand and topo IIα protein for two replicas of MD simulation; Figure S6: Dynophore model for the first replica (R1) of MD simulation of the ligand **2** in complex with ATPase domain of human topoisomerase IIα; Figure S7: Dynophore model for the third replica (R3) of MD simulation of the ligand **2** in complex with ATPase domain of human topoisomerase IIα; Figure S8: Dynophore model for the first replica (R1) of MD simulation of the ligand **TSC24** in complex with ATPase domain of human topoisomerase IIα; Figure S9: The MM/GBSA binding free energy (ΔG) for the binding of compounds **2** and **TSC24** to topo IIα; Table S8: Cancer-related drug target candidates for thiocarbohydrazone **2** identified through PharmMapper search; Table S9: ChemPLP, PLP, and PLP95 docking scores of the minimized complexes of compound **2** and the best three protein targets identified via pharmacophore similarity search; Figure S10: Binding mode and ligand interaction diagram for the binding of compound **2** to cell division protein kinase 2; Table S10: The pharmacokinetics and drug-likeness of compound **2** predicted using pkCSM and SwissADME; Table S11: The partial atomic charges of the ligand **2**; Table S12: The partial atomic charges of the ligand **TSC24**; Video S1: Dynophores_R1_compound2; Video S2: Dynophores_R2_compound2; Video S3: Dynophores_R3_compound2; Video S4: TSC24_R1_Dynophores; Video S5: TSC24_R2_Dynophores.
