# Supplementary material for: Phenotypic Discovery of Thiocarbohydrazone with Anticancer Properties and Catalytic Inhibition of Human DNA Topoisomerase IIα
Source: Pharmaceuticals (Basel). 2023 Feb 23;16(3):341. doi: 10.3390/ph16030341 (PMC10054454; doi:10.3390/ph16030341)
Supplement: Supplementary file 1 [file pharmaceuticals-16-00341-s001.zip › Supplementary material.pdf]

SUPPLEMENTARY INFORMATION

Phenotypic discovery of thiocarbohydrazones with anticancer properties and catalytic inhibition of human DNA topoisomerase IIα

Ilija N. Cvijetić[1,2], Barbara Herlah[2,3], Aleksandar Marinković[4], Andrej Perdih[2,3,*] and Snežana K. Bjelogrlić[5]

1 Faculty of Chemistry, University of Belgrade, Studentski trg 12-16, 11000 Belgrade, Serbia
2 National Institute of Chemistry, Hajdrihova 19, SI 1000 Ljubljana, Slovenia
3 Faculty of Pharmacy, University of Ljubljana, Aškerčeva 7, SI 1000 Ljubljana, Slovenia
4 Faculty of Technology and Metallurgy, University of Belgrade, Karnegijeva 4, 11120 Belgrade, Serbia
5 National Cancer Research Center, Pasterova 14, 11000 Belgrade, Serbia
* Correspondence: andrej.perdih@ki.si; Tel.: +386-1-4760-376

Table S1. Anticancer activity of thiocarbohydrazones **1**-**4**.

	**THP-1**	**MCF-7**		**AsPC-1**
**Compound**	$ApoC_{50}$ [μM]	$ApoC_{50}$ [μM]	EC_{50} [μM]	EC_{50} [μM]
**1**	56 ± 2	75 ± 5	/	/
**2**	30 ± 2	/	8 ± 4	/
**3**	11 ± 1	/	/	/
**4**	19 ± 7	35 ± 3	/	34 ± 5

The activities are expressed as $ApoC_{50}$ (concentration of compound that induces apoptosis in 50 % of treated cells) and as EC_{50} (concentration of compound that corresponds to midpoint of sigmoidal curve, computed in case investigated compound induce death in less than 50 % of treated cells).

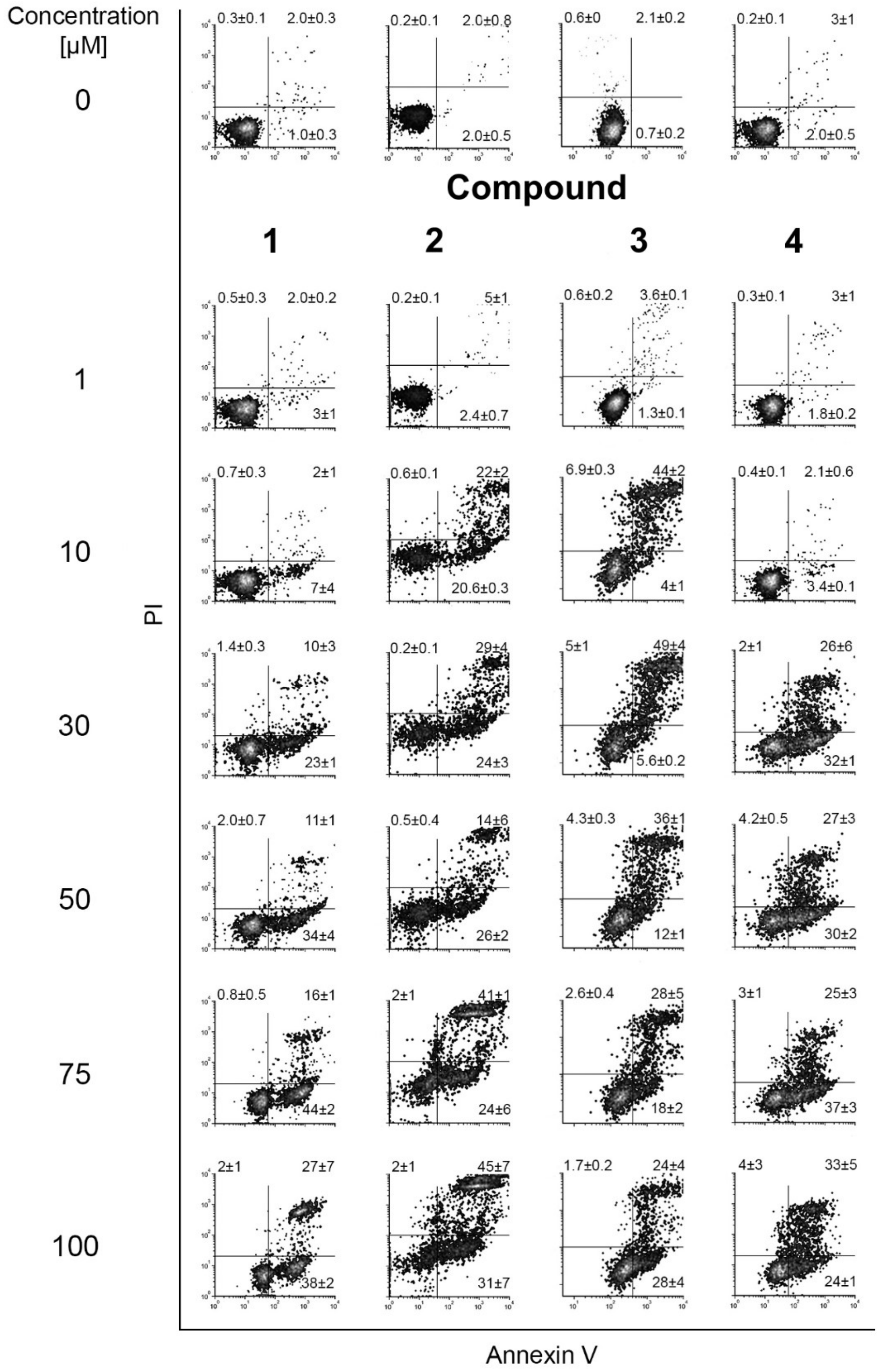

Figure S1. Types of cell death in THP-1 cells treated with investigated compounds, determined by means of Annexin V/PI dual staining assay after 24 h incubation. In Annexin V/PI dot plots cells are discriminated as viable (non-stained cells, lower left quadrant), cells in early phase of apoptotic death (Annexin V single-stained cells, lower right quadrant), cells in advanced phases of cell death (double-stained cells, upper right quadrant), and necrotic cells (PI single-stained cells, upper left quadrant). Results are represented as the mean ± SD percentages of two replicates from independent experiments.

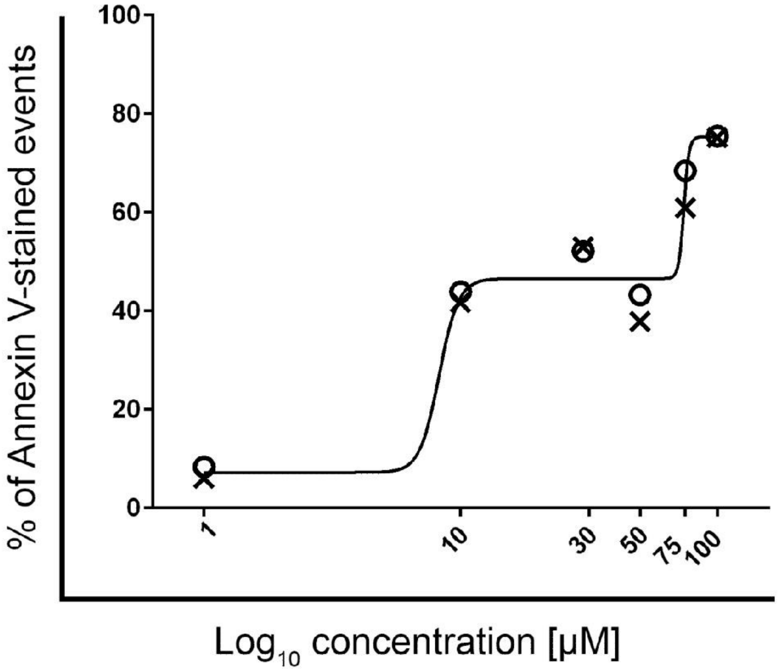

Figure S2. Concentration-response curve for **2** on THP-1 cells after 24 h treatment. Results in the graph represent summarized percentages of Annexin V single- and double-stained cells from two independent experiments (circles and crosses). Curve was computed using biphasic model for hill-shaped curve equation in GraphPad Prism 6 software.

Table S2. The results of HTS human topo IIα relaxation assay (initial screening)

Compound	1	2	3	4	Etoposide
% topo IIα inhibition at 50 μM	34	83	10	6	70

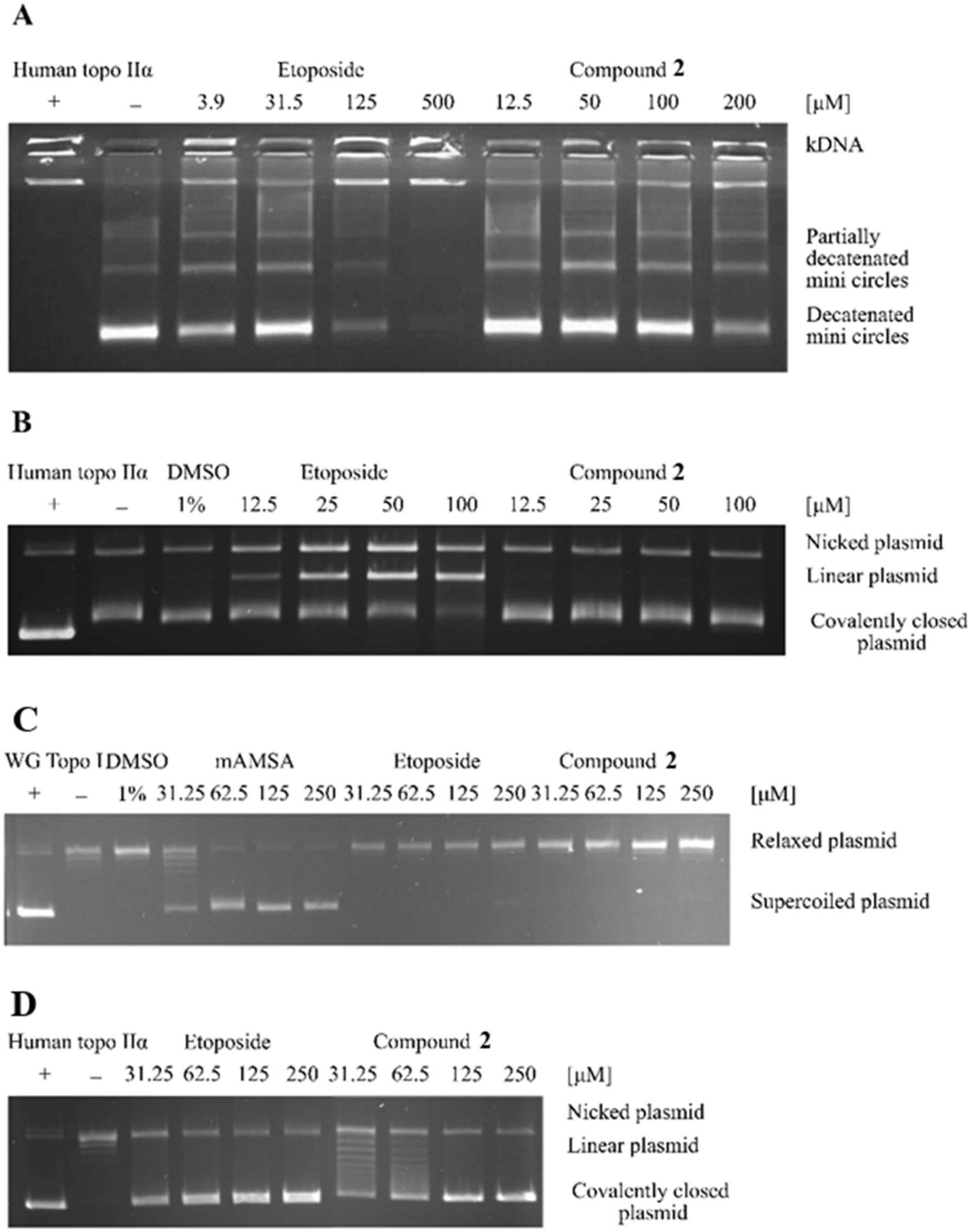

Figure S3. The results of second run of human topo IIα decatenation assay, human topo IIα cleavage assay, unwinding assay and topoisomerase IIα relaxation assay. (**A**) Results of the human topo IIα decatenation assay (second run). The assay was performed at four different concentrations of compound **2** (12.5, 50, 100 and 200 μM), and of etoposide as positive control (3.9, 31.5, 125, 500 μM); (**B**) Human topo IIα cleavage assay (second run) The assay was performed at four different concentrations of compound **2** (12.5, 25, 50 and 100 μM), and etoposide was used as a positive control (12.5, 25, 50 and 100 μM); (**C**) Unwinding assay (with relaxed substrate). The assay was performed at four different concentrations of compound **2** (31.5, 62.5, 125, 250 μM), and of the positive control intercalator mAMSA (31.5, 62.5, 125, 250 μM); (**D**) Results of the topoisomerase IIα relaxation assay (second run). The assay was performed at four different concentrations of compound **2** (31.5, 62.5, 125 and 250 μM), and etoposide as positive control (31.5, 62.5, 125 and 250 μM).

Table S3: Results of the topo IIα-mediated decatenation assay (in duplicates) for thiocarbohydrazone **2** and etoposide at different concentrations represented as the % of the decatenated kDNA.

**Compound**	**% Decatenated Assay 1**				**% Decatenated Assay 2**				**% Decatenated Average**			
	**Concentration (μM)**				**Concentration (μM)**				**Concentration (μM)**			
**[Etoposide]**	**3.9**	**31.5**	**125**	**500**	**3.9**	**31.5**	**125**	**500**	**3.9**	**31.5**	**125**	**500**
% Decat	100	89.79	38.78	7.99	100	65.11	32.71	9.58	100	77.45	35.75	8.79
**[2]**	**6.25**	**12.5**	**25**	**50**	**6.25**	**12.5**	**25**	**50**	**6.25**	**12.5**	**25**	**50**
% Decat	90.20	89.11	63.11	45.96	91.80	89.91	86.32	66.16	91.00	89.51	74.72	56.06

Table S4. Percentage of linear DNA, determined in the topo IIα-mediated cleavage assay (in duplicates) for etoposide and thiocarbohydrazone **2** at four concentrations.

**Compound**	**% Linear**	**% Linear**	**% Linear Average**
DNA alone	0	0	0
DNA + topo IIα	0	0	0
DNA + topo IIα + DMSO	0.81	0	0.81
**Etoposide** 12.5 μM	14.77	13.40	14.08
**Etoposide** 25 μM	32.39	28.65	30.52
**Etoposide** 50 μM	41.64	37.51	39.57
**Etoposide** 100 μM	43.16	50.43	46.79
**2** 12.5 μM	0.41	0.45	0.47
**2** 25 μM	0.32	0.54	0.43
**2** 50 μM	0.63	0.21	0.42
**2** 100 μM	0.00	0.04	0.4

Table S5. Results of the topo IIα-mediated relaxation assay (in duplicates) for thiocarbohydrazone **2** and etoposide at different concentrations represented as the % of the topo IIα inhibition.

**Compound**	**% Inhibition Assay 1**				**% Inhibition Assay 2**				**% Inhibition Average**			
	**Concentration (μM)**				**Concentration (μM)**				**Concentration (μM)**			
**[Etoposide]**	**31.5**	**62.5**	**125**	**250**	**31.5**	**62.5**	**125**	**250**	**31.5**	**62.5**	**125**	**250**
% Inh.	28.70	89.91	100	100	27.79	93.67	100	100	28.25	91.79	100	100
**[2]**	**31.5**	**62.5**	**125**	**250**	**31.5**	**62.5**	**125**	**250**	**31.5**	**62.5**	**125**	**250**
% Inh.	3.36	37.63	100	100	28.00	84.30	100	100	15.68	60.97	100	100

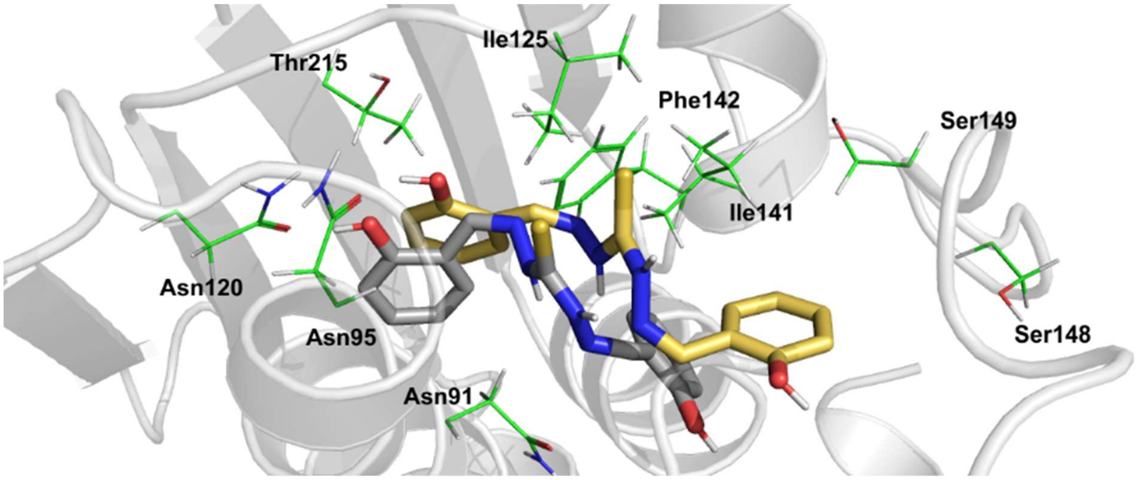

Figure S4. Two conformations of compound **2** observed in three replicas of MD simulations. Bent conformation (CF1) is depicted with grey carbons; Linear conformation (CF2) is depicted with yellow carbons.

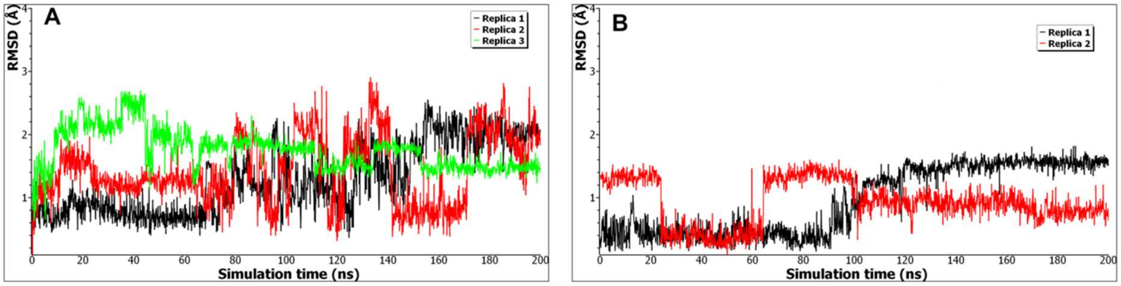

Figure S5. RMSD of the ligand heavy atoms for each replica of MD simulation of **2** (A) and **TSC24** (B) bound within ATPase domain of human topo IIα.

Table S6. Average RMSD of the ligand **2** and topo IIα protein for the three replicas of MD simulation.

	Ligand atoms	Protein backbone
	RMSD ± SD (Å)	
**Replica 1**	1.282 ± 0.557	2.011 ± 0.243
**Replica 2**	1.415 ± 0.536	2.150 ± 0.408
**Replica 3**	1.748 ± 0.322	2.493 ± 0.466

Table S7. Average RMSD of the **TSC24** ligand and topo IIα protein for two replicas of MD simulation.

	Ligand atoms	Protein backbone
	RMSD ± SD (Å)	
**Replica 1**	0.952 ± 0.536	1.662 ± 0.297
**Replica 2**	0.917 ± 0.355	1.700 ± 0.270

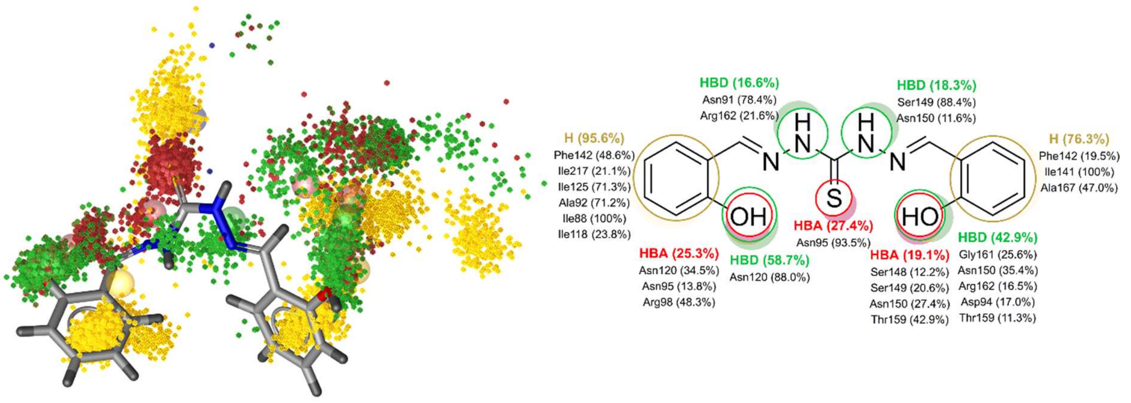

Figure S6. Dynophore model for the **first** replica (R1) of MD simulation of the ligand **2** in complex with ATPase domain of human topoisomerase IIα.

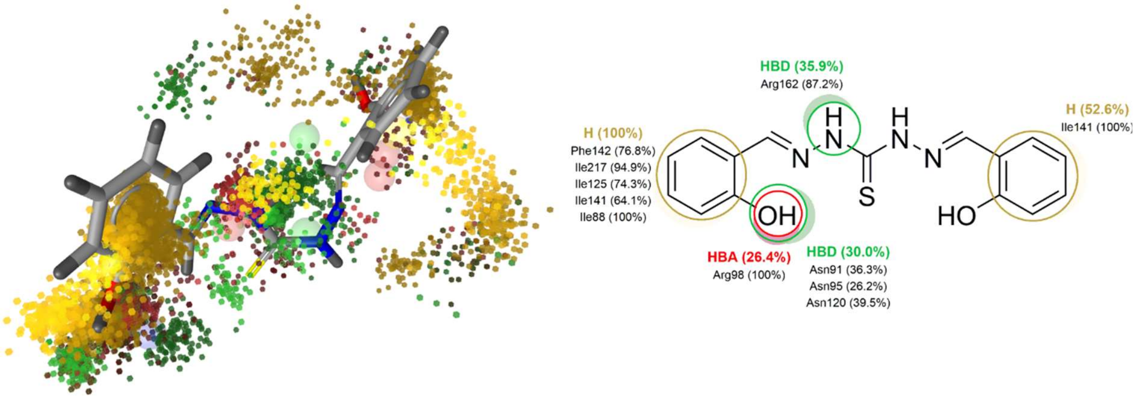

Figure S7. Dynophore model for the **third** replica (R3) of MD simulation of the ligand **2** in complex with ATPase domain of human topoisomerase IIα.

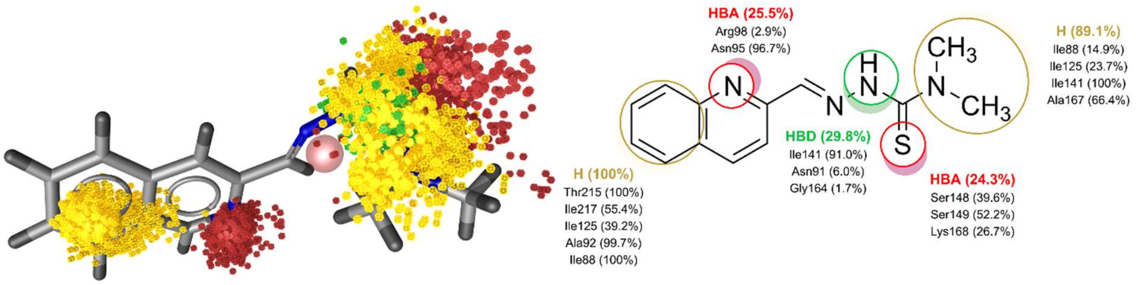

Figure S8. Dynophore model for the **first** replica (R1) of MD simulation of the ligand **TSC24** in complex with ATPase domain of human topoisomerase IIα.

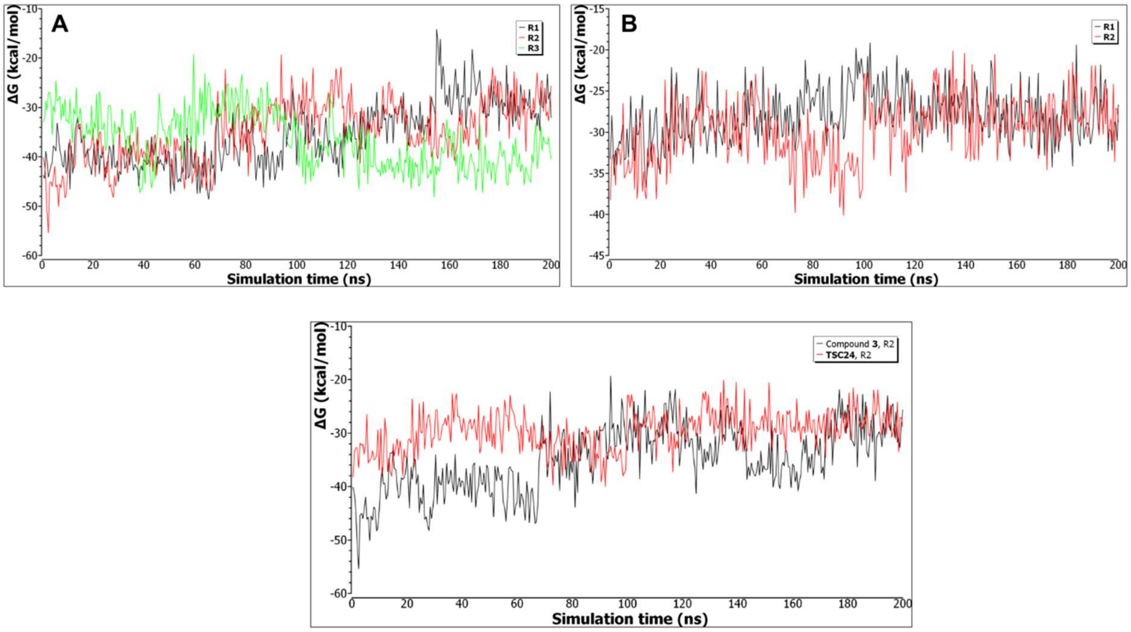

Figure S9. The MM/GBSA binding free energy (ΔG) for the binding of compounds **2** and **TSC24** to topo IIα. Calculated for 400 equidistant frames of MD simulations of (**A**) compound **2** (three replicas R1-R3) and (**B**) compound **TSC24** (two replicas R1, R2) bound to topo IIα (**C**) Comparison of MM/GBSA binding free energy (ΔG) calculated for 400 equidistant frames of MD simulations of compounds **2** (replica R2) **TSC24** (replica R2) bound to topo IIα ATP binding site.

Table S8. Cancer-related drug target candidates for the thiocarbohydrazone **2** identified through PharmMapper search.

Pharmacophore model PDB code	Normalized fit score	Z-score	Target name
1MD3	0.9616	1.3729	Glutathione S-transferase P
1JD0	0.8947	0.7580	Carbonic anhydrase 12
2VTH	0.8724	0.6633	Cell division protein kinase 2
2POW	0.8572	3.2039	Carbonic anhydrase 2
1T7V	0.7363	1.6826	Zinc-alpha-2-glycoprotein
1Q6K	0.6912	1.4285	Cathepsin K

Table S9. ChemPLP, PLP and PLP95 docking score of the minimized complexes of **2** and the best three protein targets identified by pharmacophore similarity search.

	Cell division protein kinase 2 (PDB:2VTH)	Glutathione S-transferase P (PDB: 1MD3)	Carbonic anhydrase 12 (PDB: 1JD0)
ChemPLP	-100.13	-77.08	-64.36
PLP	-90.98	-73.09	-64.58
PLP95	-146.66	-105.33	-90.58

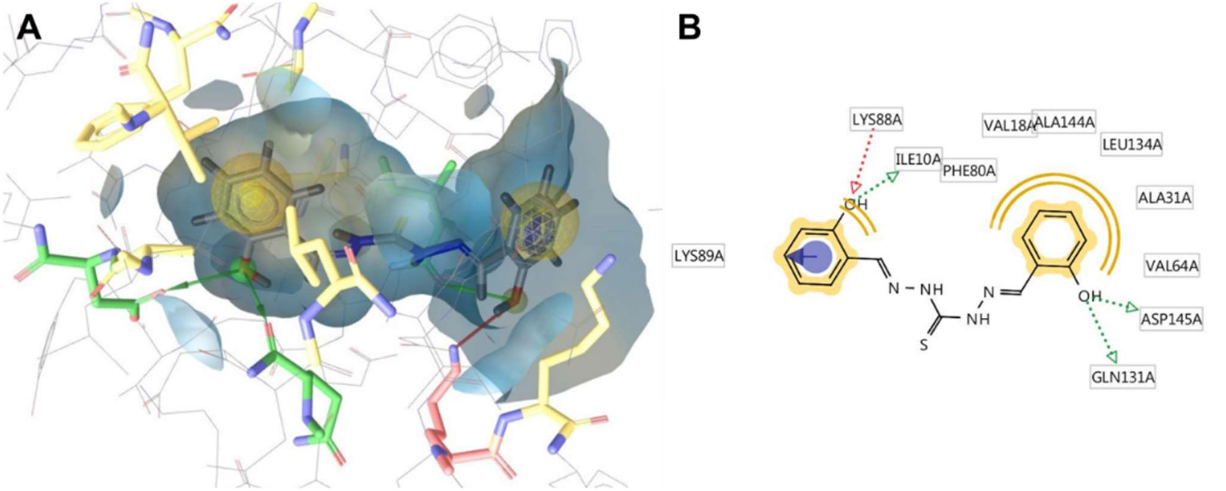

Figure S10. Binding mode and ligand interaction diagram for the binding of **2** to cell division protein kinase 2. (A) Binding mode of thiocarbohydrazone **2** into the active site of cell division protein kinase 2 (PDB: 2VTH) obtained by the combination of molecular docking and subsequent 100 ps of MD relaxation. The receptor binding site is shown as transparent surface, and the ligand interactions are color-coded (red for HBA, green for HBD and yellow for hydrophobic interactions); (B) Ligand interaction diagram showing the amino acid residues important for the stabilization of this complex as identified by LigandScout.

Table S10. The pharmacokinetics and drug-likeness of **2** predicted using pkCSM and SwissADME.

	**Descriptors**	**Value, category**
**Absorption and Distribution**	MlogP	1.63
	TPSA, Å^2	121.33
	Number of rotatable bonds	6
	ESOL Solubility (mol/L)	$4.13 \cdot 10^{-4}$, soluble
	GI Absorption (% absorbed)	90.518, High
	Log Caco-2 permeability (10^{-6}cm/s)	0.949
	BBB permeability (log BB)	-0.763, No
	CNS permeability (log PS)	-2.515
	P-gp substrate	No
	Volume of distribution (log L/kg)	0.128
	Log Kp (skin permeation, cm/s)	-6.43
**Toxicity**	AMES toxicity	No
	hERG I inhibitor	No
	hERG II inhibitor	Yes
	Hepatotoxicity	No
	Skin sensitization	No
	Minnow toxicity (log mM)	2.756
**Med. chem.**	# of Lipinski violations	0
	# of PAINS	1
	Synthetic accessibility (1-very easy to 10-very difficult)	2.75
	Leadlikeness	Yes

Table S11. The partial atomic charges and atom types of the ligand **2**.

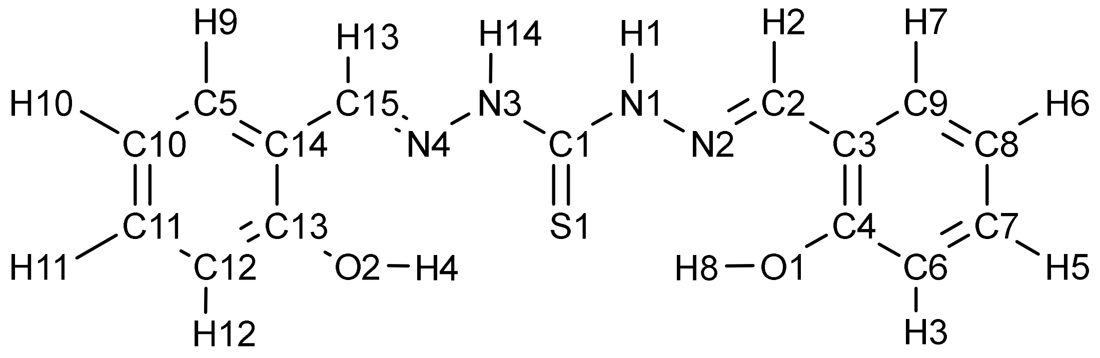

Name	Type	Q	Name	Type	q
O2	oh	-0.533	H14	hn	0.162
H4	ho	0.399	C1	c	-0.258
O1	oh	-0.533	S1	s	-0.392
H8	ho	0.399	N1	n	0.298
C13	ca	0.285	H1	hn	0.162
C12	ca	-0.294	N2	n2	-0.396
H12	ha	0.183	C2	c3	0.106
C11	ca	-0.095	H2	h4	0.096
H11	ha	0.151	C3	ca	0.020
C10	ca	-0.187	C4	ca	0.285
H10	ha	0.152	C6	ca	-0.294
C5	ca	-0.181	C7	ca	-0.095
H9	ha	0.159	C8	ca	-0.187
C14	ca	0.020	C9	ca	-0.181
C15	ce	0.106	H3	ha	0.183
H13	h4	0.096	H5	ha	0.151
N4	n2	-0.396	H6	ha	0.152
N3	n	0.298	H7	ha	0.159

Table S12. The partial atomic charges atom types of the ligand **TSC24**.

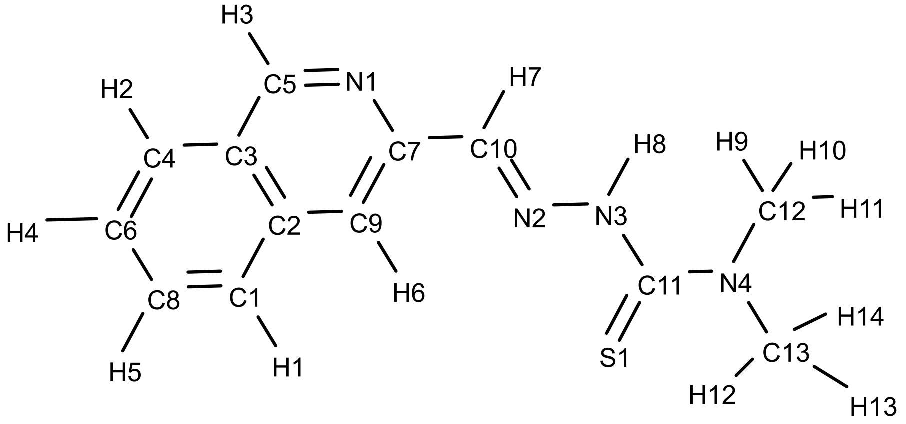

Name	Type	q	Name	Type	q
C9	ca	-0.712	C10	ce	0.062
H6	ha	0.221	H7	h4	0.130
C2	ca	0.647	N2	n2	-0.183
C1	ca	-0.438	N3	n	-0.280
H1	ha	0.210	H8	hn	0.274
C8	ca	-0.062	C11	c	0.197
H5	ha	0.144	S1	s	-0.476
C6	ca	-0.130	N4	n	0.089
H4	ha	0.141	C13	c3	-0.225
C4	ca	-0.183	H12	h1	0.099
H2	ha	0.159	H13	h1	0.099
C3	ca	-0.271	H14	h1	0.099
C5	ca	0.423	C12	c3	-0.225
H3	h4	0.063	H9	h1	0.099
N1	nb	-0.733	H10	h1	0.099
C7	ca	0.564	H11	h1	0.099
